# Clinical Efficacy of Tofacitinib in Treating Granulomatous Reaction After Mesotherapy: A Case Series Analysis

**DOI:** 10.1111/jocd.70799

**Published:** 2026-03-19

**Authors:** Chang Shu, Tao Zhang, Jia‐Wei Liu, Chen‐yu Zhu, Rou‐yu Fang, Qiu‐ning Sun, Nian Li, Feng Li

**Affiliations:** ^1^ Department of Dermatology Peking Union Medical College Hospital, Chinese Academy of Medical Sciences, and Peking Union Medical College Beijing People's Republic of China

## Abstract

**Background:**

Mesotherapy, a widely utilized minimally invasive cosmetic procedure, carries potential risks of adverse reactions due to non‐standardized protocols and overuse. Delayed granulomatous reactions represent a chronic complication, imposing significant physical and psychological burdens on patients.

**Objective:**

This case series aims to evaluate the efficacy and safety of tofacitinib in managing non‐infectious granulomatous reactions following mesotherapy.

**Methods:**

This retrospective analysis included six patients diagnosed with non‐infectious granulomatous reactions post‐mesotherapy, treated at Peking Union Medical College Hospital between October 2021 and April 2025. All patients received oral tofacitinib. Clinical outcomes, treatment regimens, and safety profiles were assessed.

**Results:**

All six patients demonstrated significant improvement in skin lesion severity, with no treatment‐related adverse events observed during follow‐up.

**Conclusion:**

Oral tofacitinib exhibits promising clinical efficacy and a favorable safety profile for non‐infectious granulomatous reactions induced by mesotherapy, positioning it as a viable therapeutic option during the inflammatory phase.

## 1 Introduction

Mesotherapy involves placement of substances into the “mesoderm” (dermis and subcutis), such as water, nutrients, and bioactive agents. Through fine needle injection, these components penetrate the skin barrier and enter the superficial dermis, serving to enhance hydration, deliver therapeutic effects, and promote cellular rejuvenation [1, 2, 3]. Hyaluronic acid, a common component, augments dermal hydration and stimulates collagen and elastin synthesis. Despite its generally favorable safety profile, mesotherapy is associated with adverse reactions due to non‐standardized procedures, limited approved injectable materials in mainland China, and a propensity for overuse. Early complications include infections (predominantly nontuberculous mycobacteria) [4], while delayed reactions encompass nodules, granulomas, panniculitis, fat necrosis, lichenoid eruptions, and hypersensitivity responses. Notably, hyaluronic acid, as a foreign body, may trigger diverse inflammatory responses, with non‐infectious granulomatous reactions increasingly reported [5]. These manifest as persistent papules and nodules at injection sites, lasting months to years. The etiology remains incompletely elucidated but may involve impurities in injected materials, hyaluronic acid degradation products, and individual variability in immune responses.

Current therapeutic strategies for non‐infectious granulomatous inflammation lack consensus, relying primarily on topical and systemic corticosteroids and immunosuppressants. Prolonged treatment durations heighten the risk of adverse effects, including Cushingoid features, gastric ulcers, osteoporosis, skin atrophy, telangiectasia, hirsutism, and opportunistic infections. Tofacitinib, a Janus kinase (JAK) inhibitor targeting the JAK/STAT pathway, has shown efficacy in cutaneous granulomatous disorders. This retrospective case series evaluates the clinical efficacy and safety of tofacitinib for mesotherapy‐induced noninfectious granulomatous reactions.

## 2 Methods

This retrospective case series study was documented by the Department of Dermatology of Peking Union Medical College Hospital. All patients provided written informed consent for data collection and publication.

### 2.1 Patients

Six patients diagnosed with non‐infectious granulomatous reactions following mesotherapy, treated at Peking Union Medical College Hospital from October 2021 to April 2025, were included. Initial evaluations occurred at the Departments of Dermatology and Plastic Surgery. All presented with persistent skin eruptions at mesotherapy injection sites and received oral tofacitinib. Comprehensive laboratory tests, imaging studies, and, where applicable, skin biopsies excluded infections, malignancies, and other dermatologic conditions. Diagnoses were confirmed by two consultant dermatologists.

### 2.2 Assessments

Detailed clinical histories and available photographs were reviewed, documenting clinical presentations, laboratory results (complete blood count, comprehensive metabolic panel, T‐SPOT.TB, autoantibodies profile), percentage of facial area involved, initial diagnosis, prior treatments, complications, disease duration before admission, time to onset of skin eruptions post‐mesotherapy, and time to clinical improvement following tofacitinib initiation.

Lesion location and morphology were described by two dermatologists. Dermoscopy and high‐frequency ultrasound findings were recorded when available. Skin biopsies, when performed, were evaluated by dermatopathologists. Treatment‐related adverse events were monitored throughout follow‐up.

### 2.3 Outcome Measures

Complete remission was defined as the complete disappearance of clinical symptoms. Partial remission was defined as a significant improvement in skin lesions with residual papules, hyperpigmentation, or minimal erythema. Treatment failure was defined as persistent or worsening symptoms despite at least 4 weeks of therapy.

### 2.4 Case 1

A 32‐year‐old female developed red papules at all injection sites three days post‐mesotherapy with a product named “Cytocare 532,” primarily containing hyaluronic acid. Lesions were occasionally pruritic. At an external facility, she received two monthly intramuscular injections of Diprospan (betamethasone propionate 5 mg and betamethasone sodium phosphate 2 mg per mL), with transient improvement but relapse upon discontinuation. Oral thalidomide improved symptoms but was discontinued due to intolerable numbness of extremities and constipation. Subsequent treatment with oral prednisone (30 mg/day) and cyclosporine (100 mg/day) reduced some papules and nodules, but complete resolution was not achieved after three months.

At our department, laboratory tests (complete blood count, urinalysis, comprehensive metabolic panel, autoantibody profile, infection panel, T‐SPOT.TB) were unremarkable. Dermoscopy revealed orange‐yellow homogeneous areas with peripheral blurred vascular dilatation. High‐frequency ultrasound showed scattered hypoechoic areas in the superficial dermis. Histopathology indicated epithelioid and lymphohistiocytic infiltration in the superficial dermis, suggestive of granulomatous inflammation, with no amorphous hyaluronic acid deposits on H&E staining.

**FIGURE 1:**
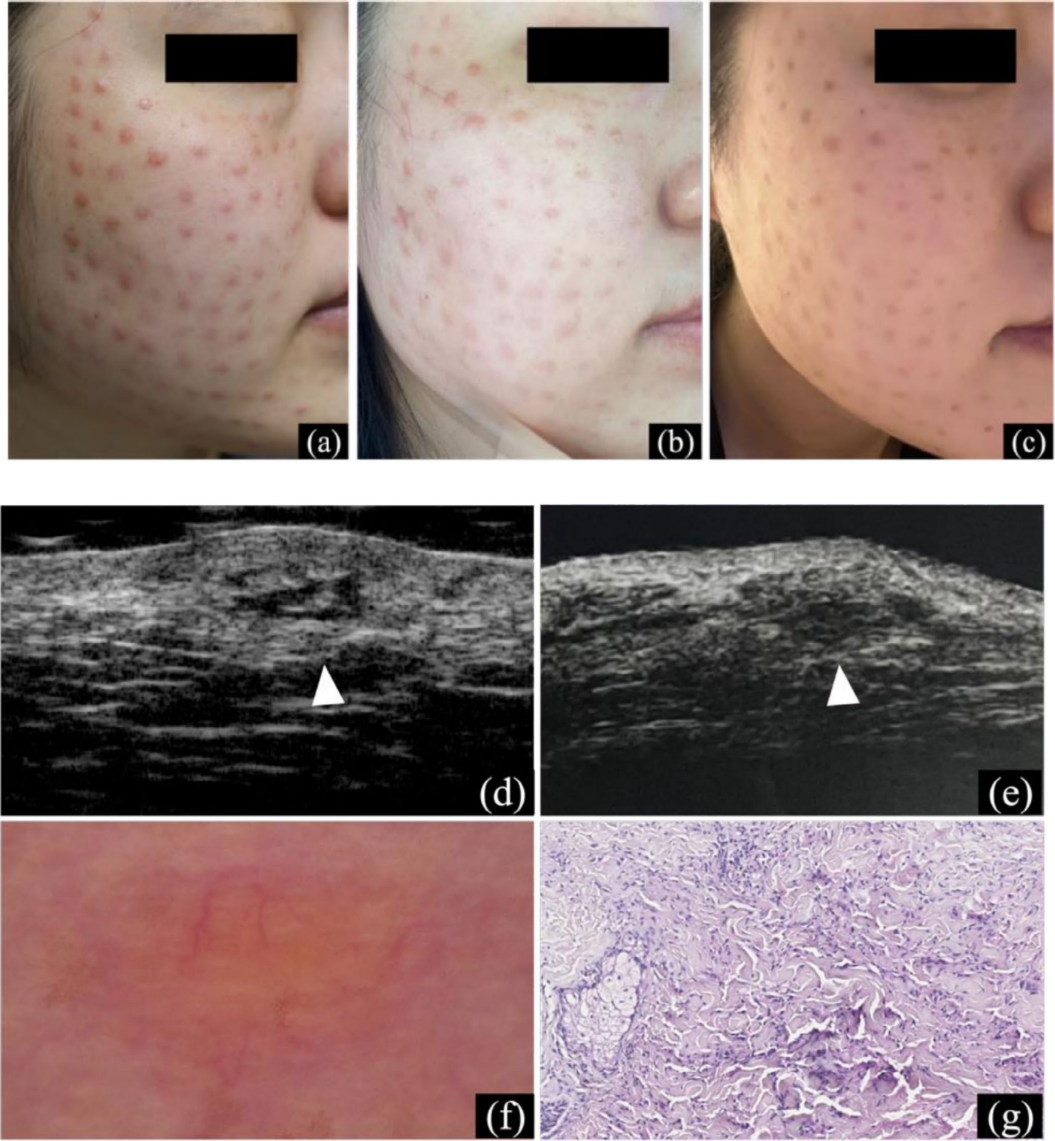
Skin manifestations of Case 1. (a) Multiple red papules on the face at initial visit; (b) Four weeks post‐intralesional adalimumab; (c) Marked improvement after 4 months of oral tofacitinib; (d, e) Skin ultrasound at initial visit and after 8 months (50 MHz), white arrow indicating the target lesion area; (f, g) Dermoscopy (×10) and histopathology (H&E, ×200) of the target lesion at initial visit.

Initial treatment involved intralesional adalimumab (80 mg every 2 weeks, twice), yielding slight lesion reduction but no significant improvement. Therapy was switched to oral tofacitinib (10 mg/day), resulting in marked symptom improvement early on. After one month, nodules stabilized without further regression. Tofacitinib was continued for four months and discontinued, with no recurrence observed three months later (Figure 1).

### 2.5 Case 2

A 40‐year‐old female presented with persistent, asymptomatic red papules following facial mesotherapy with a mixture of hyaluronic acid, nucleotides, and vitamins. At an external facility, she received one intramuscular Diprospan injection and two monthly intralesional Diprospan injections, with improvement but relapse upon discontinuation. Laboratory tests at our hospital (complete blood count, comprehensive metabolic panel, T‐SPOT.TB) were unremarkable.

**FIGURE 2:**
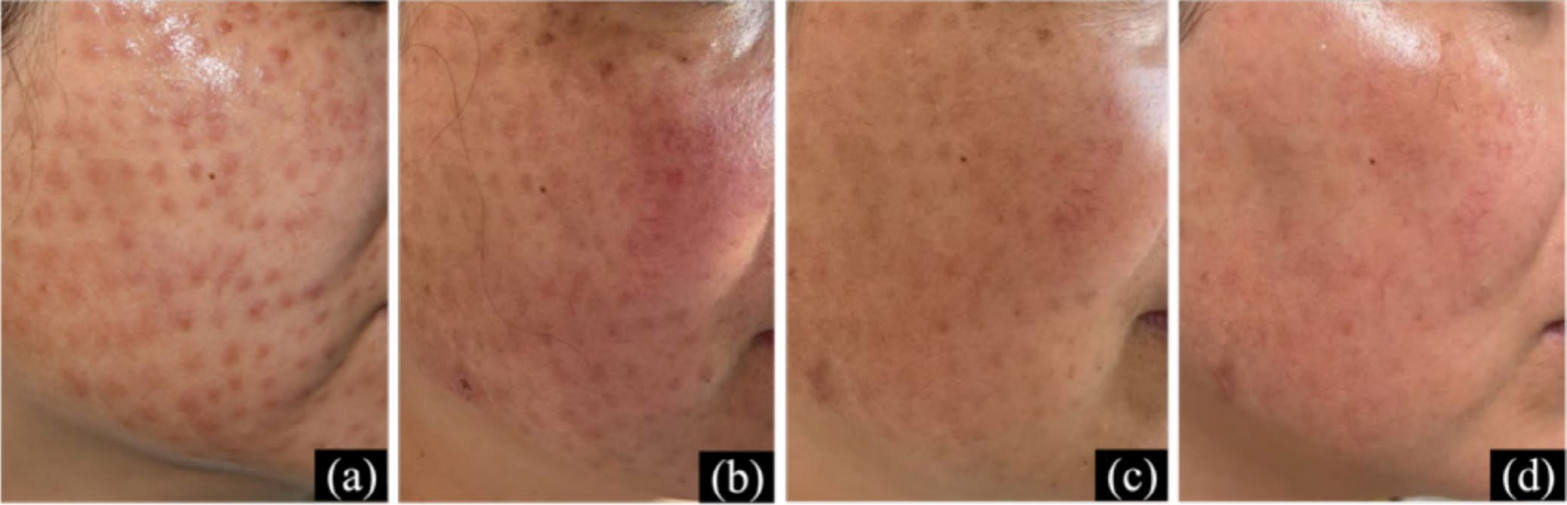
Skin manifestations of Case 2. (a) At initial visit; (b) After 2 weeks of treatment; (c) After 6 weeks of treatment; (d) After 14 weeks of treatment.

Treatment included oral minocycline (50 mg twice daily) and prednisone (40 mg/day) for 2 weeks, with symptom improvement. To minimize corticosteroid‐related adverse effects, upadacitinib (15 mg/day) was added, with prednisone tapered by 5 mg weekly. Lesions continued to improve without recurrence. Due to cost, treatment was adjusted to tofacitinib (5 mg/day) after 2 weeks, with prednisone gradually discontinued. No recurrence was observed, and follow‐up is ongoing (Figure 2).

### 2.6 Case 3

A 36‐year‐old female developed papules and pruritus at injection sites two months post‐mesotherapy with hyaluronic acid and collagen. Oral minocycline and topical mometasone furoate cream for over 2 weeks yielded slight improvement, but symptoms relapsed upon discontinuation. Laboratory tests (complete blood count, comprehensive metabolic panel, T‐SPOT.TB) were normal. Dermoscopy showed reddish‐brown homogeneous areas with peripheral blurred vascular dilatation. High‐frequency ultrasound revealed scattered hypoechoic areas in the superficial dermis with vascular dilatation.

**FIGURE 3:**
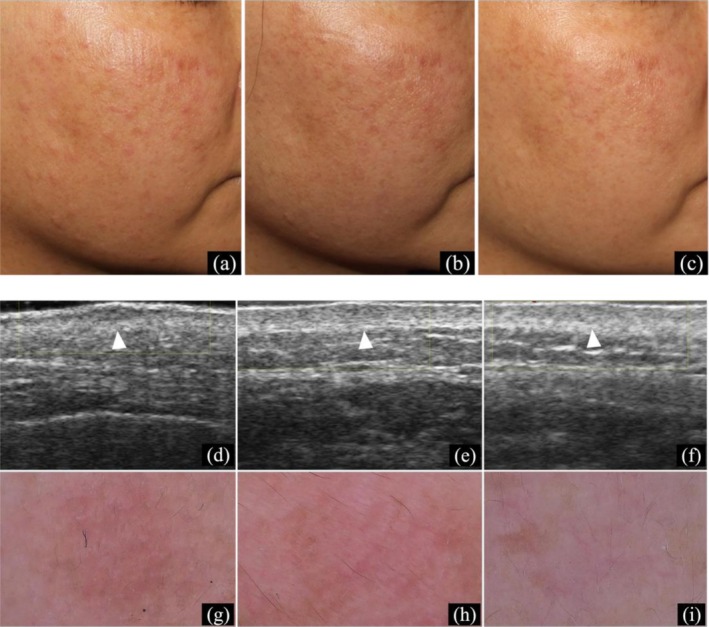
Skin manifestations of Case 3. (a–c) Papules on the cheek at 0, 2, and 6 weeks post‐treatment; (d–f) Skin ultrasound at 0, 2, and 6 weeks (50 MHz), white arrow indicating the target lesion area; (g–i) Dermoscopy at 0, 2, and 6 weeks (×10).

Treatment with oral tofacitinib (10 mg/day) and topical 0.1% tacrolimus ointment resulted in significant regression of papules and nodules within 2 weeks. Tofacitinib was reduced to 5 mg/day for 4 weeks, with a stable skin condition. The patient remains on treatment and under follow‐up (Figure 3).

### 2.7 Case 4

**FIGURE 4:**
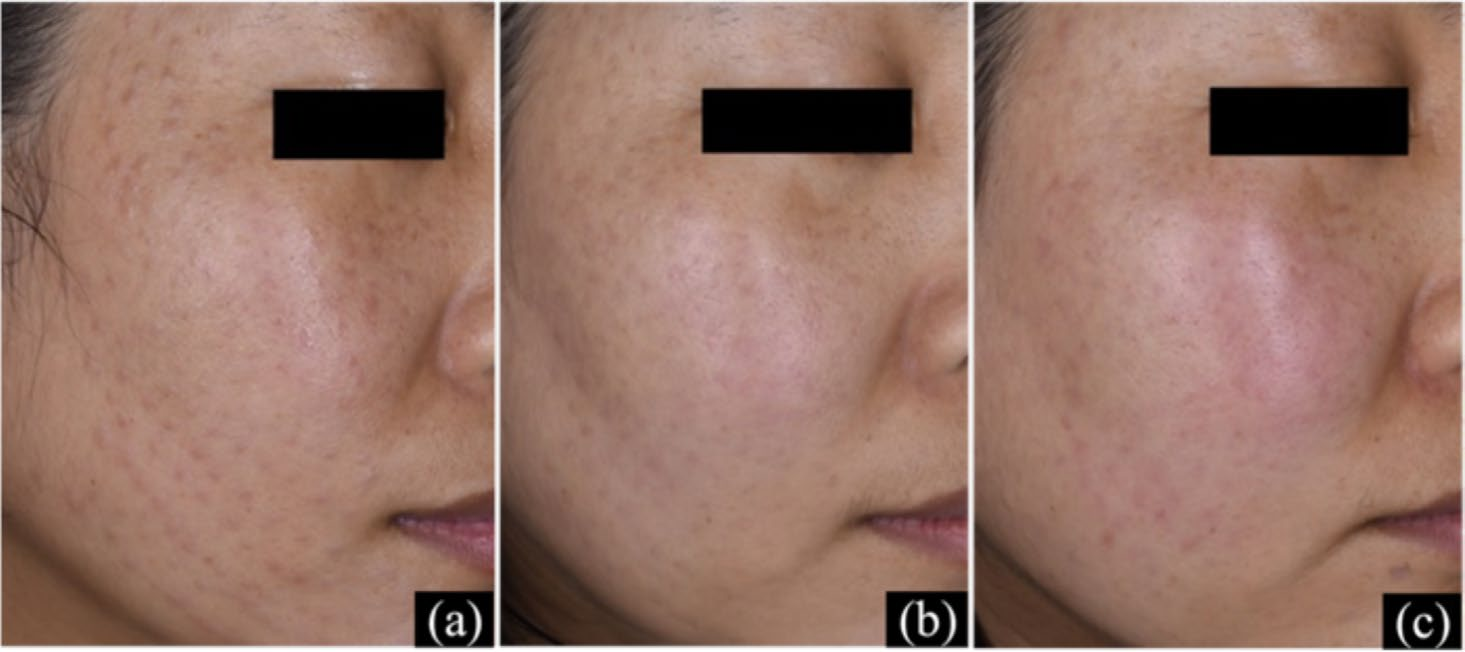
Skin manifestations of Case 4. (a–c) Papules on the cheek at 0, 8, and 16 weeks post‐treatment.

A 32‐year‐old female developed erythema, multiple papules, and pruritus five days post‐mesotherapy with a mixture of hyaluronic acid, nucleotides, vitamins, and botulinum toxin. At our hospital, one intramuscular Diprospan injection and oral methylprednisolone (16 mg/day) improved symptoms within one week, but severe hand tremors prompted a reduction of methylprednisolone to 8 mg/day. At 8 weeks, a Cushingoid appearance was evident. Oral tofacitinib (5 mg/day) was added, and methylprednisolone was discontinued, resulting in continued improvement (Figure 4).

### 2.8 Case 5

**FIGURE 5:**
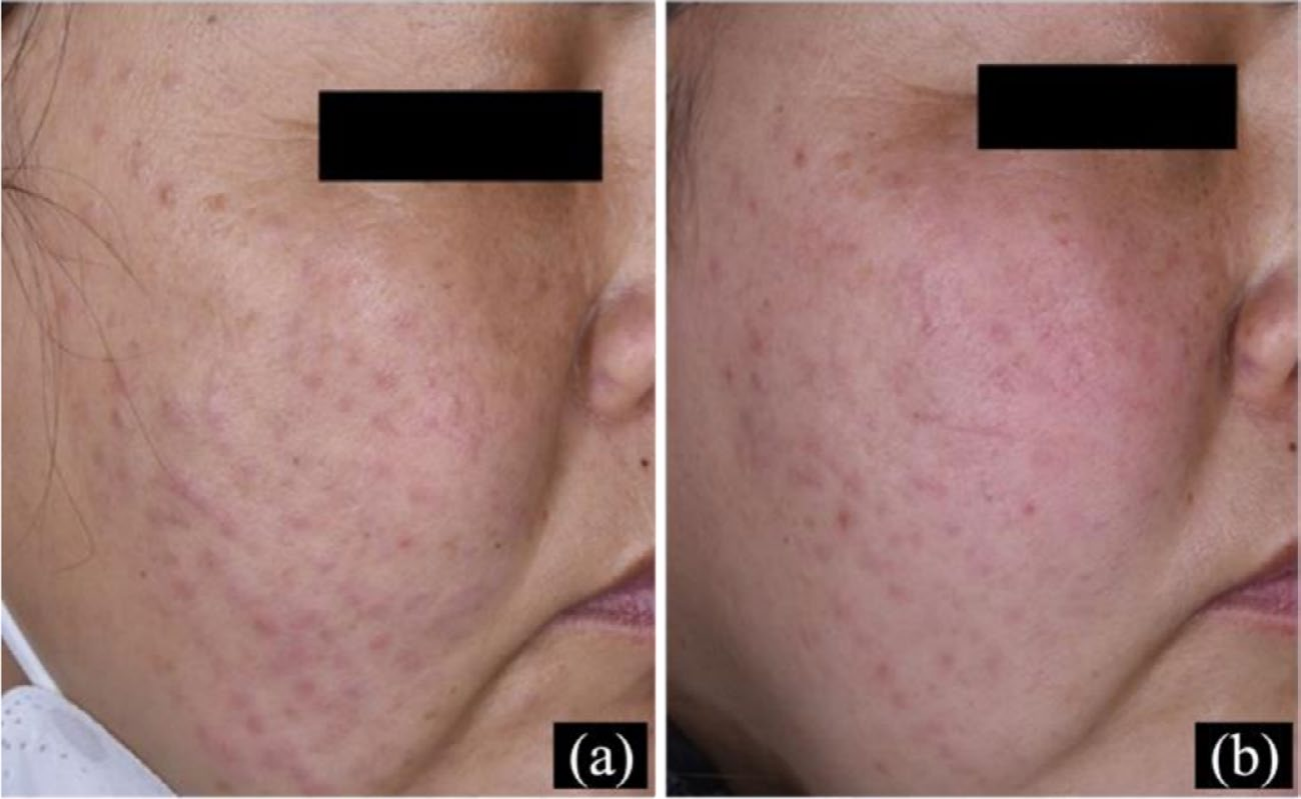
Skin manifestations of Case 5. (a) At the initial visit, (b) After 20 weeks of treatment.

A 39‐year‐old female developed facial papules three days post‐mesotherapy with unknown components. One intramuscular Diprospan injection improved symptoms within one week, but acid reflux, palpitations, and headaches emerged. At 4 weeks, treatment was adjusted to oral methylprednisolone (16 mg/day) and tofacitinib (5 mg/day), with gradual improvement (Figure 5).

### 2.9 Case 6

**FIGURE 6:**
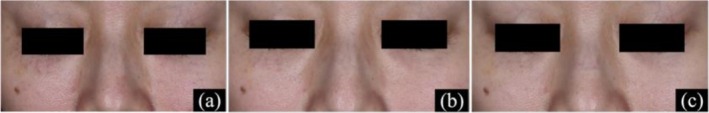
Skin manifestations of Case 6. (a–c) Periorbital papules at 0, 2, and 6 weeks of treatment.

A 38‐year‐old female developed infiltrative red papules, prominent in the periorbital area, three days post‐mesotherapy with recombinant hyaluronic acid and human collagen. Initial treatment included one intramuscular Diprospan injection and oral clarithromycin (250 mg twice daily). At 2 weeks, lesions improved. To reduce corticosteroid use, oral tofacitinib (5 mg/day) was added, combined with intralesional triamcinolone and pulsed dye laser therapy, yielding significant improvement (Figure 6).

## 3 Results

In this study, six patients were diagnosed with non‐infectious granulomas. Each patient received oral tofacitinib, some of them combined with systemic corticosteroids and other treatments. All patients demonstrated marked improvement. No severe adverse effects were observed during the treatment of tofacitinib. The summary of patient information and assessments is presented in Table 1.

**TABLE 1:** Summary of patient information and assessments.

Case	Age/Sex	Mesotherapy Components	Time to Onset	Initial Symptoms	Previous Treatments	Diagnostic Methods	Tofacitinib Regimen	Treatment Duration	Clinical Outcome	Adverse Events
1	32/F	Hyaluronic acid	3 days	Red papules at all injection sites, occasional itching	IM Diprospan (2×), oral thalidomide, oral prednisone 30 mg/d + cyclosporine 100 mg/d	Dermoscopy, high‐frequency ultrasound, histopathology	10 mg/d for 4 months	4 months	Significant improvement, no recurrence 3 months post‐discontinuation	None reported
2	40/F	Hyaluronic acid, nucleotides, vitamins	Persistence of injection papules	Persistent papules, no pain/itching	IM Diprospan (1×), intralesional corticosteroids (2×)	Clinical evaluation	Initial treatment: Minocycline 50 mg bid + prednisone 40 mg/d for 2 weeks, followed by upadacitinib 15 mg/d for 2 weeks, then tofacitinib 5 mg/d	Ongoing at time of report	Progressive improvement, no recurrence	None reported
3	36/F	Hyaluronic acid, collagen	2 months	Papules and itching at injection sites	Oral minocycline, topical mometasone furoate cream	Dermoscopy, high‐frequency ultrasound	10 mg/d for 1 week, then 5 mg/d	Ongoing at time of report	Significant improvement after 1 week, stable condition	None reported
4	32/F	Hyaluronic acid, nucleotides, vitamins, botulinum toxin	5 days	Persistent injection papules, local redness, swelling, itching	IM Diprospan (1×), oral methylprednisolone 16 mg/d	Clinical evaluation	5 mg/d with concurrent methylprednisolone taper	Not specified	Continued improvement	Hand tremors (attributed to methylprednisolone), Cushingoid appearance
5	39/F	Unknown components	3 days	Facial papules	IM Diprospan (1×)	Clinical evaluation	5 mg/d with concurrent methylprednisolone 16 mg/d	Not specified	Gradual improvement	Acid reflux, palpitations, headache (attributed to corticosteroids)
6	38/F	Recombinant human collagen, hyaluronic acid	3 days	Infiltrative red papules, prominent in periorbital area	IM Diprospan (1×), oral clarithromycin 250 mg bid	Clinical evaluation	5 mg/d, later combined with intralesional triamcinolone injections and pulsed dye laser	Not specified	Further improvement	None reported

## 4 Discussion

Among non‐infectious complications of mesotherapy, delayed granulomatous reactions are a significant concern. Initially associated with injections of bile salts, choline, carnitine, and silicon dioxide, these reactions have increased with the widespread use of non‐cross‐linked hyaluronic acid in mesotherapy [1, 6, 7].

The mechanism of granulomatous reactions to non‐cross‐linked hyaluronic acid is not fully elucidated. As most hyaluronic acid is produced via bacterial fermentation, impurities such as proteins, bacterial endotoxins, and cell wall components (e.g., lipoteichoic acid, LTA) may trigger inflammation [8]. Low‐molecular‐weight (LMW) hyaluronic acid fragments may be recognized as tissue damage signals, activating macrophages and inducing inflammation, with reported delayed nodule rates of 1%–4.25% [9]. Superficial injection of non‐cross‐linked hyaluronic acid containing LMW fragments may be captured by antigen‐presenting cells, precipitating granulomatous reactions.

In our six cases, the onset of this granulomatous reaction was beyond three days post‐injection, and persistence for over three months if it was not treated. Histopathology revealed dermal infiltration of lymphocytes, histiocytes, and macrophages, with granuloma formation, suggestive of a type IVa delayed‐type hypersensitivity (sarcoidal) reaction. Immunohistochemical studies support monocyte–macrophage activation as a key driver [10, 11]. Symptom severity may correlate with individual immune variability, injected material type, injection volume, and depth.

Type IVa hypersensitivity involves epithelioid histiocytes, lymphocytes, and occasional multinucleated Langhans‐type giant cells, mediated by TH1 cells and cytokines such as IFN‐γ, TNF‐α, and IL‐6. Therefore, the following potential treatment methods can be inferred from the pathogenesis:

### 4.1 Local Treatment

Intralesional corticosteroids, including triamcinolone and betamethasone, are the most preferred therapy for foreign body granulomas, particularly filler‐induced, due to their non‐selective anti‐inflammatory effects on lymphocytes, macrophages, and fibroblasts [12, 13]. Bleomycin and 5‐fluorouracil have also been reported as effective [9, 12]. Given the numerous injection sites and small nodule size in mesotherapy, the risk of skin atrophy from excessive injection warrants caution. Topical calcineurin inhibitors may aid in controlling superficial granuloma inflammation.

### 4.2 Systemic Treatment

Systemic corticosteroids are employed for recurrent granulomas, with initial doses of at least 30 mg/day to prevent relapse [14]. Minocycline, alone or combined with corticosteroids, has shown efficacy [5]. Other agents, including colchicine, cyclosporine, methotrexate, thalidomide, hydroxychloroquine, isotretinoin, allopurinol, and JAK inhibitors (including tofacitinib, baricitinib, upadacitinib, abrocitinib), have been reported [14, 15, 16, 17].

JAK inhibitors, such as tofacitinib, exert non‐selective anti‐inflammatory effects on lymphocytes, akin to corticosteroids, but are better suited for long‐term use to avoid systemic corticosteroid side effects. They block IFN‐γ‐mediated inflammation, a critical driver of granuloma formation, making them promising for cutaneous granulomatous disorders [16, 17, 18]. In our cohort, all patients responded well to tofacitinib, with good tolerability and mild adverse effects. A de‐escalation approach was employed: Tofacitinib was initiated at 10 mg/day, reduced to 5 mg/day upon significant symptom relief, and continued for 3–6 months to ensure inflammation control and granuloma resolution. Monitoring for opportunistic infections, liver enzyme abnormalities, lipid profiles, and coagulation indices is essential during long‐term therapy.

### 4.3 Surgical and Physical Therapy

Granuloma excision is not first‐line due to infiltrative growth and indistinct boundaries, risking infection and poor wound healing. Cryotherapy, fractional laser, microneedle radiofrequency, and chemical peels may improve fibrotic nodules as late‐stage alternatives [19, 20]. Hyperbaric oxygen therapy has shown benefit in individual cases [21].

It is worth mentioning that one of our patients trialed intralesional adalimumab, with a suboptimal response, possibly due to insufficient dosage or duration. Prior reports suggest anti‐TNF therapy can disrupt granulomas and is effective in sarcoidosis, granuloma annulare, necrobiosis lipoidica, and inflammatory bowel disease‐related skin lesions [22, 23]. Systemic anti‐TNF agents may be considered for select patients.

### 4.4 Prevention

Strict control of mesotherapy indications and avoidance of misuse are critical. Injectable materials should be rigorously screened, with high‐risk agents further evaluated. For individuals with a high risk of allergic reaction, a skin test may be conducted to confirm tolerance before proceeding with treatment, such as a patch test or intradermal test on the forearm.

## 5 Conclusion

In conclusion, the risk–benefit balance of mesotherapy must be carefully weighed. Though rare, delayed granulomatous reactions cause facial disfigurement and significantly impact the quality of life. Vigilance and prompt management of these complications are essential for practitioners in aesthetic medicine.

## Author Contributions

Chang Shu provided cases, conducted patient follow‐up, organized the data, and wrote the manuscript. Qiu‐ning Sun and Feng Li provided cases and conducted patient follow‐up. Nian Li performed biopsies and photographic documentation for some of the cases. Tao Zhang, Jia‐Wei Liu, Chen‐yu Zhu, and Rou‐yu Fang provided valuable suggestions for this study and participated in the revision of the manuscript.

## Funding

This study was supported by Beijing Key Clinical Specialty Construction Project and National Key Clinical Specialty Project of China.

## Ethics Statement

This paper ensures the protection of patient privacy and security. This retrospective case series study was documented by the Department of Dermatology of Peking Union Medical College Hospital. All patients provided written informed consent for data collection and publication.

## Consent

Informed consent was obtained from all individual participants included in the study. The authors affirm that human research participants provided informed consent to publish the images.

## Conflicts of Interest

The authors declare no conflicts of interest.

## Data Availability

The data that support the findings of this study are available on request from the corresponding author. The data are not publicly available due to privacy or ethical restrictions.
